# Plant and Microbial Responses to Repeated Cu(OH)_2_ Nanopesticide Exposures Under Different Fertilization Levels in an Agro-Ecosystem

**DOI:** 10.3389/fmicb.2018.01769

**Published:** 2018-07-31

**Authors:** Marie Simonin, Benjamin P. Colman, Weiyi Tang, Jonathan D. Judy, Steven M. Anderson, Christina M. Bergemann, Jennifer D. Rocca, Jason M. Unrine, Nicolas Cassar, Emily S. Bernhardt

**Affiliations:** ^1^Center for the Environmental Implications of Nanotechnology, Duke University, Durham, NC, United States; ^2^Department of Biology, Duke University, Durham, NC, United States; ^3^Department of Ecosystem and Conservation Sciences, University of Montana, Missoula, MT, United States; ^4^Division of Earth and Ocean Sciences, Nicholas School of the Environment, Duke University, Durham, NC, United States; ^5^Soil and Water Sciences Department, University of Florida, Gainesville, FL, United States; ^6^Department of Plant and Soil Sciences, University of Kentucky, Lexington, KY, United States

**Keywords:** copper hydroxide, nanomaterials, pasture, microbial extracellular enzyme activities, terrestrial mesocosms, mycorrhizal colonization, fungicide, nitrogen fixation

## Abstract

The environmental fate and potential impacts of nanopesticides on agroecosystems under realistic agricultural conditions are poorly understood. As a result, the benefits and risks of these novel formulations compared to the conventional products are currently unclear. Here, we examined the effects of repeated realistic exposures of the Cu(OH)_2_ nanopesticide, Kocide 3000, on simulated agricultural pastureland in an outdoor mesocosm experiment over 1 year. The Kocide applications were performed alongside three different mineral fertilization levels (Ambient, Low, and High) to assess the environmental impacts of this nanopesticide under low-input or conventional farming scenarios. The effects of Kocide over time were monitored on forage biomass, plant mineral nutrient content, plant-associated non-target microorganisms (i.e., N-fixing bacteria or mycorrhizal fungi) and six soil microbial enzyme activities. We observed that three sequential Kocide applications had no negative effects on forage biomass, root mycorrhizal colonization or soil nitrogen fixation rates. In the Low and High fertilization treatments, we observed a significant increase in aboveground plant biomass after the second Kocide exposure (+14% and +27%, respectively). Soil microbial enzyme activities were significantly reduced in the short-term after the first exposure (day 15) in the Ambient (-28% to -82%) and Low fertilization (-25% to -47%) but not in the High fertilization treatment. However, 2 months later, enzyme activities were similar across treatments and were either unresponsive or responded positively to subsequent Kocide additions. There appeared to be some long-term effects of Kocide exposure, as 6 months after the last Kocide exposure (day 365), both beta-glucosidase (-57% in Ambient and -40% in High fertilization) and phosphatase activities (-47% in Ambient fertilization) were significantly reduced in the mesocosms exposed to the nanopesticide. These results suggest that when used in conventional farming with high fertilization rates, Kocide applications did not lead to marked adverse effects on forage biomass production and key plant–microorganism interactions over a growing season. However, in the context of low-input organic farming for which this nanopesticide is approved, Kocide applications may have some unintended detrimental effects on microbially mediated soil processes involved in carbon and phosphorus cycling.

## Introduction

Novel applications of nanotechnology for plant protection and nutrition is leading to the development of so-called “nanopesticides” and “nanofertilizers.” The emergence of these nano-enabled agrochemicals may be a promising avenue for reducing agricultural impacts on the environment and on human health (Kah, 2015; Liu and Lal, 2015). Because nanomaterial-enabled pesticides and fertilizers are being optimized for longer, sustained release it has been assumed that they will be more effective at lower application rates and may have fewer environmental consequences (Mishra et al., 2017). However, the limited knowledge on the environmental fate and potential impacts of these nano-agrochemicals currently hampers our ability to assess the true benefits and risks of these new formulations compared to conventional pesticides and fertilizers (Benelli et al., 2017; Kah et al., 2018).

The direct, intentional, and repeated application of nano-agrochemicals could potentially become a pathway by which large masses of nanomaterials may be introduced into agroecosystems. Previous ecotoxicological studies raised concerns about the impact of nanomaterials on plant health and soil organisms (reviewed by McKee and Filser, 2016; Tripathi et al., 2017) and even food quality and soil fertility (Priester et al., 2012; Chen et al., 2015; Judy et al., 2015). However, most studies are currently performed under simplified laboratory conditions (e.g., soil microcosms or hydroponics), at unrealistically high concentrations, or use pristine nanoparticles that are not comparable to commercial nano-enabled products (Simonin and Richaume, 2015; Chen et al., 2017). Furthermore, several parameters deserve to be investigated in more depth, such as plant–microbial interactions, fluctuating environmental conditions, physiological acclimation and evolutionary adaptations to repeated exposures, or interactive effects with other stressors that may be strong drivers of the environmental fate and ecotoxicity of nano-agrochemicals (Cornelis et al., 2014; McKee and Filser, 2016). As the production and release of these novel nano-formulations in agro-ecosystems may increase in the future, developing realistic long-term environmental assessments of nano-agrochemical impacts is imperative.

Integrating nanotechnology in agriculture is still in its infancy but several products are already commercialized such as copper-based (Cu) nanopesticides used as a fungicide and bactericide. The commercial pesticide Kocide 3000^®^ contains nanoparticles and micron-sized particles of Cu, and nanosheets composed of Cu(OH)_2_ and thus this agrochemical product containing nanomaterials as the active ingredient is considered as a nanopesticide (Adeleye et al., 2014). It is approved for organic crop production and can be applied to a wide variety of crops including forage crops, vegetables, fruits, and trees. The product is suspended in water and sprayed on the plants to prevent the development of a variety of fungal and bacterial diseases. Its antifungal and antibacterial properties emerge mainly from the sustained release of Cu ions following the dissolution of the Cu(OH)_2_ particles (Keller et al., 2017). While this nanopesticide may protect the crops from fungal and bacterial diseases, it may have unintended consequences on non-target plant-associated microorganisms involved in plant nutrition, such as mycorrhizal communities or nitrogen fixing bacteria (Hussain et al., 2009). Moreover, beneficial soil microorganisms that degrade organic matter (OM), which maintain long-term soil fertility, may be sensitive to this broad-spectrum antimicrobial product (Bünemann et al., 2006; Lejon et al., 2008). If so, these nanopesticide applications might have unintended consequences for soil fertility and plant yields over the long term, especially as a result of repeated exposures.

Nanopesticides are typically added to agricultural soils or crops alongside other agrochemicals that can interact to increase (i.e., additive interactions) or decrease (i.e., antagonistic interactions) the potential effects on organisms. In particular, agro-ecosystems are exposed to different fertilization regimes depending on the farming type (e.g., conventional or organic), tillage practices, and the type of crop grown. Fertilization could indirectly influence Cu bioavailability as a consequence of the changes in soil pH and OM (Leita et al., 1999). Additionally, fertilization might influence the plant or microbial physiological response to the nanopesticide (Conway et al., 2015). Agroecologists have suggested that microbial community resilience will increase with greater resource availability (De Vries and Shade, 2013), while communities already stressed by nutrient limitation may have less energy to cope with additional stressors, like a pesticide (Griffiths and Philippot, 2013).

In this context, we asked three main questions: (1) Do repeated applications of a Cu(OH)_2_ nanopesticide impact plant biomass, plant-associated non-target microorganisms (i.e., N-fixing bacteria or mycorrhizal fungi), and soil microorganisms involved in OM degradation in an agro-ecosystem?, (2) How does fertilization influence the Cu(OH)_2_ nanopesticide effects on the biotic endpoints?, and (3) What are the legacy effects of repeated application of a Cu(OH)_2_ nanopesticide on agro-ecosystem functioning? To address these questions, we conducted a 1-year outdoor mesocosm experiment where we exposed forage crop cover to a series of repeated applications of the commercially available Cu(OH)_2_ nanopesticide (Kocide 3000), simulating realistic agricultural application rates. This experiment was performed under three fertilization levels (Ambient, Low, and High) to test for interactive effects between the nanopesticide and enhanced resource availability of fertilization additions and simulate relevant farming practices with varying levels of nutrient inputs (e.g., conventional or organic farming). The mesocosms were sampled on short- (15 days) and long-term (2.5 months) timescales after the nanopesticide exposures to assess the resistance and resilience of the agro-ecosystem to this disturbance. A final sampling was performed 6 months after the last Kocide exposure (1 year after the initiation of the treatments) to assess the legacy effects of this nanopesticide after a growing season.

## Materials and Methods

### Experimental Design

The outdoor terrestrial mesocosms were set up in the Duke Forest (36°00’57.3”N 78°58’49.8”W, Durham, NC, United States). Each mesocosm [51 cm (l) × 25 cm (w) × 5 cm (h)] was filled with ∼81 kg of a sandy-clay-loam soil (Sands and Soils, Durham, NC, United States) comprised of: 57.7% sand, 20.5% clay, 21.9% silt, and 4% organic matter (pH = 5.8). The weather conditions at the mesocosm site were monitored during the entire experiment (see Supplementary Figure S1 for air temperature and precipitation data).

The mesocosms were seeded with seven forage crops plant species, selected based on growth performance and common agricultural use in pasture regions of the surrounding North Carolina Piedmont (Miguel et al., 2014): *Trifolium pratense* (legume, Fabaceae), *Chamaecrista fasciculata* (legume, Fabaceae), *Brassica napus* (annual forb, Brassicaceae), *Cichorium intybus* (invasive perennial forb, Asteraceae), *Sorghastrum nutans* (native perennial graminoid, Poaceae), and *Urochloa ramosa* (perennial graminoid, Poaceae). In the spring of 2016, each box was mowed and additionally seeded with *Medicago sativa* (legume, Fabaceae). The different plant species were grouped in three main plant functional groups: forbs, graminoids, and legumes.

To control water availability within the mesocosms, a sprinkler system was installed in the summer, to water each mesocosm every 3 days for 15 min unless a rain event occurred.

Three fertilization treatments were initiated on September 4, 2015, 8 months before the beginning of the nanopesticide applications. Fertilization levels (Ambient, Low, and High) were adjusted using the Osmocote^®^ fertilizer (The Scotts Company, Marysville, OH, United States) following the NCDA recommended rates for pasture crops^1^. The Ambient mesocosms received no supplemental fertilizer; the Low mesocosms received 1.72 g N, 0.77 g P, and 1.29 g K; and the High mesocosms received 3 × Low (5.15 g N, 2.32 g P, 3.86 g K). Before the Kocide exposures started, the soil chemical characteristics in the different fertilization levels were determined (**Table 1**). The soil NH_4_^+^ and NO_3_^-^ concentrations were determined after a KCl extraction (2M) on a Lachat QuikChem 8000 (Lachat Instruments, Milwaukee WI, United States). Soil pH was measured according to ISO 10390 in pure water and soil OM matter was determined by loss on ignition.

**Table 1:** Soil chemical characteristics for the three fertilization levels prior to the initiation of the nanopesticide exposures.

Fertilization	pH	NO_3_^-^ (μg N-NO_3_^-^/g dry soil)	NH_4_^+^ (μg N-NH_4_^+^/g dry soil)	OM content (%)
Ambient	6.02 ± 0.25	1.63 ± 1.0	1.56 ± 0.7	4.32 ± 0.27
Low	6.20 ± 0.18	7.53 ± 4.0	11.99 ± 4.0	4.22 ± 0.01
High	5.17 ± 0.25	19.09 ± 5.3	38.20 ± 8.4	3.89 ± 0.03


**FIGURE 1:**
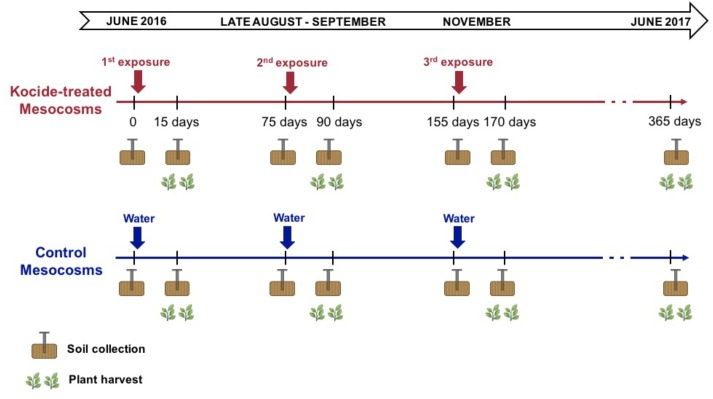
Timeline of the experiment presenting the timing of the treatment applications and the sampling performed (soil cores and plant harvest).

On June 8th, 2016, the Kocide^®^ 3000 (DuPont^TM^, Wilmington, DE, United States) nanopesticide treatment regimes started. Kocide contains Cu(OH)_2_ nanoparticles (Adeleye et al., 2014) with an average primary particle size of 38.7 ± 8.2 nm (TEM) and an average hydrodynamic diameter of 120 ± 30 nm in the dosing water with a secondary peak with particles size greater than 700 nm (Simonin et al., 2018). The Cu content in Kocide is 26.5 ± 0.9%, while other elements (e.g., C, O, Na, Al, Si, P, S, and Zn) account for 73.5% of the dry mass of the product. We sprayed (Hudson, Model 13581, Chicago, IL, United States) the foliage of each mesocosm with the Kocide suspension (6.68 mg/L in DI water) so the aboveground plant biomass exposure was 30 mg/m^2^, per the manufacturer’s instructions for dosage and exposure mode. Kocide applications were performed 15 days before each subsequent plant harvest (on days 0, 75, and 155 of the experiment, **Figure 1**). The control mesocosms were sprayed with the same volume of deionized water to hold constant across mesocosms the changes in soil moisture availability and temporary cooling from the spraying. Following the recommendations of the North Carolina Cooperative Extension Service for forage crops, three plant biomass harvests were conducted at a 2.5-month interval (on days 15, 90, and 170 of the experiment, **Figure 1**). The six treatments combinations were replicated across six independent mesocosms for a total of 36 mesocosms. In summary, the mesocosms were exposed to 3 consecutive nanopesticide exposures or to control exposures during the experiment under three different fertilization conditions: 2 nanopesticide conditions (including control) × 3 fertilization treatments × 6 replicates = 36 mesocosms.

### Soil Measurements

#### Soil Sampling and Characterization

Small soil cores (2 cm diameter, 0–7 cm depth) were collected before each new nanopesticide exposure (day 0, 75, 155) and 15 days after the exposure right before the plant harvest (day 15, 90, 170). An additional soil collection was performed 365 days after the first nanopesticide exposure (**Figure 1**). To avoid resampling the same spot or unevenly sampling the mesocosms, each soil core was extracted at different locations each time following a randomized sampling strategy established at the beginning of the experiment. The soil core samples were immediately stored at 4°C before analysis (less than a week). The soil samples were homogenized and sieved to <2-mm mesh (USDA standard). Soil moisture was determined by drying a ∼10 g subset of each soil core for 48 h at 105°C. Soils collected after the third, final exposure (day 170) and on the long-term sampling (day 365) were oven-dried and ground for microwave assisted acid digestion using 10:1 HNO_3_:H_2_O_2,_ following US EPA Method 3052. Total Cu concentrations were then measured using ICP-MS (7500cx, Agilent Technologies, Santa Clara, CA, United States) following US EPA method 6020A.

On these two sampling dates (day 170 and 365), an additional larger soil core (5 cm diameter, 0–15 cm depth) was collected to perform measurements of N_2_ fixation rates and root mycorrhizal colonization (detailed below).

#### Microbial Extracellular Enzyme Activities Targeting OM Degradation

The potential activity of six microbial extracellular enzymes were measured using the protocol described by Bell et al. (2013), including three C-degrading enzymes (alpha-glucosidase, beta-glucosidase and cellulase), a *N*-degrading enzyme (chitinase – *N*-acetylglucosaminidase), a P-degrading enzyme (alkaline phosphatase) and a S-degrading enzyme (arylsulfatase). Fresh soil (2.75 g) was blended with 91 mL of a sodium acetate buffer (50 mM, pH adjusted to soil pH) to obtain a homogeneous soil slurry. The soil slurry was then pipetted in 96-well deepwell plates with one of the six fluorescently labeled substrates or with fluorescent standards [4-methylumbelliferone (MUB)]. The plates were incubated for 3 h in the dark at 20°C, then centrifuged (2 min at 1000 × *g*) to pellet light-interfering soil particles, and the supernatant was transferred in black optical 96-well plates for fluorescence measurements on a plate reader (Fluostar Optima, BMG Labtech, Cary, NC, United States). Enzyme activities were quantified based on the fluorescence measured in each sample (indirect assessment of substrate degradation and fluorophore release due to enzyme activity) and the conversion of the data based on the standard dilution curves of each substrate from the MUB wells.

#### Nitrogen Fixation Rates

During the two final sampling dates (day 170 and 365), soil N_2_ fixation rates (includes both free-living and plant-associated N_2_ fixers) were measured on intact soil cores extracted from the same location in all the mesocosms. We determined N_2_ fixation rates by measuring acetylene reduction to ethylene using the standardized method of Acetylene Reduction Assays by Cavity ring-down laser Absorption Spectroscopy (ARACAS; Cassar et al., 2012). Each soil sample was incubated for 30 min in a 500 mL Erlenmeyer flask with 10% of the headspace replaced with acetylene gas. The headspace was circulated with a diaphragm pump to a cavity ring-down spectrometer (CRDS) and back to the incubation chamber in a closed loop to measure ethylene concentration at high frequency (every few seconds) and continuously. N_2_ fixation rate was determined from the rate of ethylene increase using a standard 4:1 conversion factor (Hardy et al., 1968). We recognize the quantitative uncertainty associated with the conversion factor (Visser et al., 1994), but it should not affect the qualitative comparison among different experiments. Finally, the N_2_ fixation rate was normalized to per gram of dry soil. More methodological details are available in Cassar et al. (2012).

### Plant Measurements

The aboveground plant biomass was harvested four times during the experiment (**Figure 1**): 15 days after each of the three Kocide exposures; and at the end of the experiment (day 365). At each harvest, the plant biomass was mowed with cordless grass shears, and the different plant species were sorted before measuring plant dry biomass (72 h at 60°C). Representative composite samples of the aboveground plant biomass collected on day 170 and 365 were digested using HNO_3_:H_2_O_2_ and total Cu, iron (Fe), manganese (Mn), and zinc (Zn) concentrations were then measured using ICP-MS as described above.

Using the same soil cores to estimated N_2_ fixation (day 170 and 365), root mycorrhizal colonization was determined by carefully separating the roots and mycorrhizal hyphae from the soil cores, the roots collected were stored in 50% ethanol. Later, roots were removed from the 50% ethanol, rinsed with tap water and covered with 10% (w/v) potassium hydroxide. Then, roots were placed into an autoclave where they were heated for 3 min at 121°C. The potassium hydroxide solution was removed and the roots were rinsed with tap water. Roots were then acidified by soaking them in 1.0% HCl for 5 min. After removing the HCl, roots were covered with 0.05% tryptan blue (C_34_H_28_N_6_O_14_S_4_) and stained overnight at room temperature. Tryptan blue was then removed and the roots transferred to 50% ethanol after rinsing with tap water. Mycorrhizal colonization of stained roots was then assessed via the root slide technique (Smith and Read, 2010).

### Statistical Analyses

All the endpoints were analyzed using generalized linear mixed-effects to model the effects of nanopesticide exposure (control, nanopesticide-exposed), fertilization (Ambient, Low, and High) and the interaction between nanopesticide exposure and fertilization by day of the experiment (repeated measurements: all the dates included in the model). In these models, main effects and interactions were nested by day, and mesocosm was treated as a random effect to account for serial correlation among observations from the same mesocosms over time (Zuur et al., 2009). The models were fit following a framework similar to the one described in King et al. (2016) using the *glmer* function of the lme4 package in R 3.2.3 (R Core Team, 2015). *Post hoc* comparisons were performed using the *lsmeans* function/package in R, which adjusts *p*-values to compensate for multiple comparisons, to determine significant differences between control and nanopesticide exposure conditions for each fertilization level at the different dates. A principal component analysis was performed on the soil microbial enzyme activities (*n* = 6 activities) on the data collected on day 170 and 365 for which we had the highest number of plant and soil variables measured. Using the *envfit* function in the *vegan* package in R, we tested if these plant and soil variables were significantly correlated to the enzyme activity profiles on the ordination.

## Results

### Effects of Nanopesticide Exposures on Plant Biomass

**FIGURE 2:**
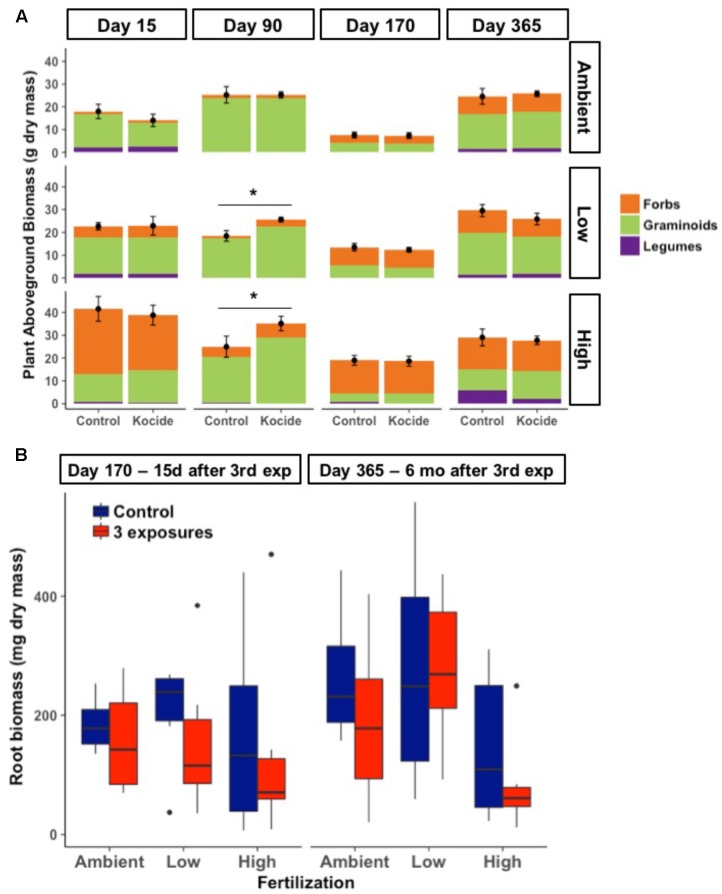
**(A)** Effects of Kocide on aboveground plant biomass among the fertilization treatments (top-bottom: Ambient, Low, and High) horizontally displayed over time. Each plant functional group (Forbs, Graminoids, and Legumes) are presented as stacked bars of respective dry biomass. The black symbols represent the mean of the plant aboveground biomass and the black lines represent the standard errors associated. Significant effects of the Kocide treatment on aboveground biomass compared to the controls are indicated by asterisks (^∗^*p* < 0.05). **(B)** Effect of Kocide exposures in the different fertilization treatments on the dry root biomass collected on day 170 and 365. The data are presented as box plots where the black horizontal line in the middle of the box represents the median and the two ends of the vertical line indicates the minimum and maximum values. The black dots represent the outliers.

Aboveground plant biomass increased significantly between each level of fertilization (*p* < 0.001); while the addition of Kocide did not significantly affect biomass production at any level of fertilization (*p* = 0.13 independent effect and *p* = 0.82 interaction effect; **Figure 2A**). Although there was no overall effect, *post hoc* tests indicated that Kocide application increased the aboveground plant biomass after the second exposure (day 90) in both the Low (14%, *p* = 0.04, **Figure 2A**) and High fertilization treatments (27%, *p* = 0.04, **Figure 2**). For the two occasions on which destructive sampling allowed us to estimate belowground root biomass (days 160 and 365), we found no effect of Kocide on belowground biomass (**Figure 2B**).

While our fertilization treatments led to shifts in plant communities, there was no consistent effect of Kocide on the relative dominance of the three-different plant functional groups (**Figure 2A**). Graminoids tended to dominate in the Ambient and Low fertilization treatments, while forbs tended to dominate in the High fertilization treatment at most dates. The single date on which we observed a Kocide treatment effect on a plant functional group was on day 90, when forb biomass was significantly increased in the mesocosms treated with Kocide in the Low fertilization condition (+62%, *p* = 0.01).

### Lack of Effects of Nanopesticide Exposures on Plant-Associated Microorganism

**FIGURE 3:**
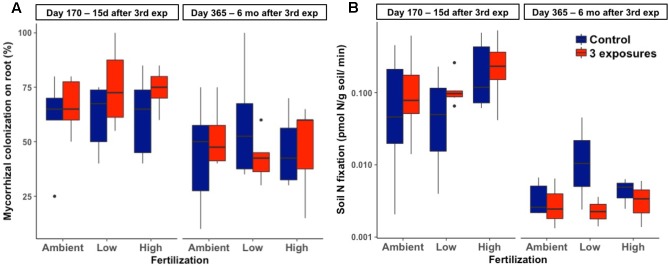
Effects of Kocide exposures and fertilizer treatments on the two final harvest days (170 and 365) on: **(A)** mycorrhizal colonization of roots (%), and **(B)** soil N_2_ fixation rate (*y*-axis on a log scale). The data are presented as box plots where the black horizontal line in the middle of the box represents the median and the two ends of the vertical line indicates the minimum and maximum values. The black dots represent the outliers.

There was no effect of Kocide exposure on the extent of mycorrhizal colonization of plant roots (*p* = 0.19, **Figure 3A**). This endpoint was also unresponsive to either fertilization (*p* = 0.95) or the interactive effects between Kocide and fertilization (*p* = 0.55). Similarly, no effect of the treatments was observed on soil N_2_ fixation rates (Kocide: *p* = 0.43, Fertilization: *p* = 0.24, Kocide × Fertilization: *p* = 0.99, **Figure 3B**). Note that on day 365, N_2_ fixation rates were low enough in some samples to be below detection limit (0.001 pmol/g soil/min, **Figure 3B**).

### Short-Term and Long-Term Effects of Repeated Applications of Nanopesticides on Soil Microbial Enzyme Activities

**FIGURE 4:**
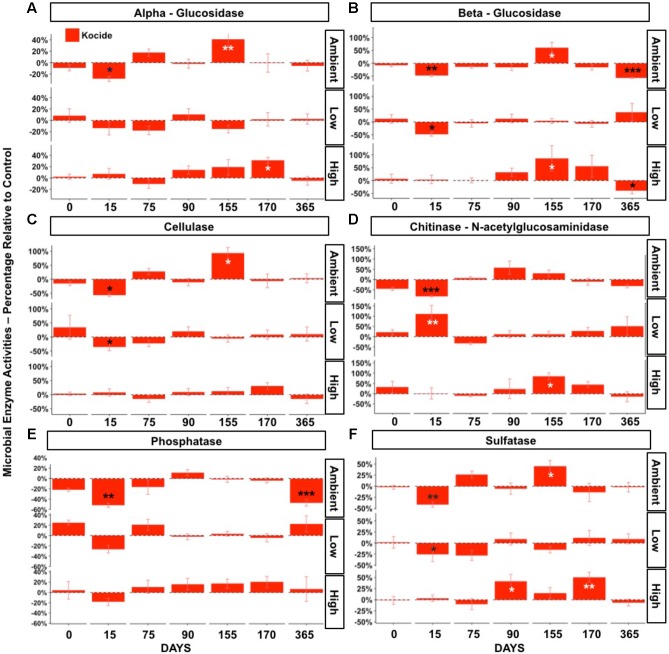
Effects of Kocide on extracellular enzyme activities in the three fertilization treatments at the seven sampling dates: (top-bottom rows): Ambient, Low, High fertilization levels. **(A)** alpha-glucosidase, **(B)** beta-glucosidase, **(C)** cellulase, **(D)** chitinase (b-1,4-*N*-acetylglucosaminidase), **(E)** alkaline phosphatase, **(F)** sulfatase. The results are presented as percentage relative to controls. Significant effects of the Kocide treatment relative to controls indicated by asterisks: ^∗^*p* < 0.05; ^∗∗^*p* < 0.01; ^∗∗∗^*p* < 0.001. The black asterisks represent significant decreases in enzyme activities, while white asterisks represent significant increases. (Note the *y*-axis scale is different on each panel).

The effects of the Kocide on potential microbial extracellular enzyme activities were strongly influenced by the fertilization level. Significant interactive effects of Kocide exposure and fertilization were observed in all six extracellular enzymes activities measured (*p* < 0.05, **Figure 4**). In the Ambient fertilization conditions, large reductions in microbial enzyme activities were observed 15 days after the first Kocide exposure (**Figure 4**). The chitinase activity exhibited the highest reduction (-82%, **Figure 4D**), followed by cellulase (-57%, **Figure 4C**), phosphatase (-52%, **Figure 4E**), beta-glucosidase (48%, **Figure 4B**), sulfatase (-41%, **Figure 4F**), and alpha-glucosidase activity (-28%, **Figure 4A**). By day 75, there were no treatment effects on enzyme activities, and enzyme activities did not decline in response to the second and third Kocide exposures. Prior to the Kocide addition on day 155 three C- degrading enzymes and sulfatase activity were substantially higher in the Kocide treated soils (**Figure 4B**). At the end of the experiment (day 365), we measured significant reductions in both beta-glucosidase and phosphatase activities in mesocosms exposed to the nanopesticides (**Figure 4B**: -57% and **Figure 4E**: -47%, respectively).

In the Low fertilization treatment, microbial enzyme activities were also altered by the first Kocide exposure (day 15), albeit to a lesser extent than in the Ambient fertilization treatment (**Figure 4**). On day 15, three enzyme activities were significantly decreased (beta-glucosidase -47%, cellulase -36%, sulfatase -25%) and the chitinase activity was stimulated by Kocide (+111%). On day 75, all the enzyme activities recovered from this initial exposure and no significant effects of the Kocide additions were observed for the remainder of the experiment at this fertilization level.

In the High fertilization treatment, Kocide treatments had limited negative effects on microbial enzyme activities and mainly generated significant augmentations after the second and third exposures. On day 90 and 170, the sulfatase activity was increased following Kocide exposures (+42% and 51%, respectively). Stimulation of the chitinase activity (+84%, **Figure 4D**) and beta-glucosidase (+87%, **Figure 4B**) were observed on day 155 and alpha-glucosidase was increased on day 170 (+31%, **Figure 4A**). Under High fertilization, only beta-glucosidase activity was negatively impacted by Kocide after 6 months (-40%, **Figure 4B**).

**FIGURE 5:**
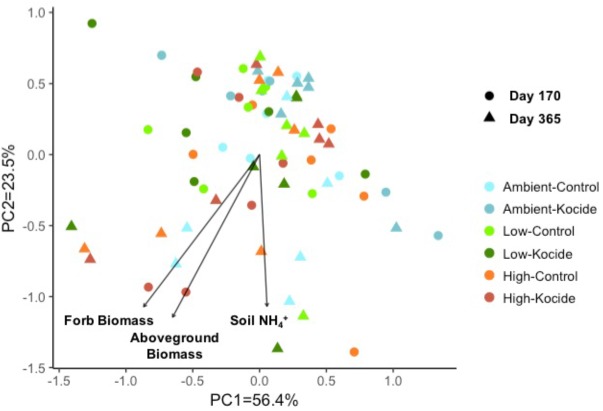
Principal component analysis (PCA) of the six soil microbial extracellular enzyme activities measured on day 170 and day 365 of the experiment. The environmental variables found to be significantly correlated to the distribution of the enzyme activity data in the ordination are represented as vectors.

Using a principal component analysis, we explored how the Kocide and fertilization treatments affected the soil enzyme activity profiles on day 170 and 365 and which environmental parameters influenced these microbial activities (**Figure 5**). This visualization did not reveal any clear shift in the enzyme activity profiles induced by our treatments but a Permanova analysis indicated a significant effect of the date of collection (*p* = 0.008) and a significant interaction between the Kocide and Fertilization treatments (*p* = 0.03). The soil NH_4_^+^, the plant aboveground biomass and forb biomass were found to be significantly correlated to the enzyme profiles on the ordination (**Figure 5**).

### Copper Accumulation in Plants and Soils

**FIGURE 6:**
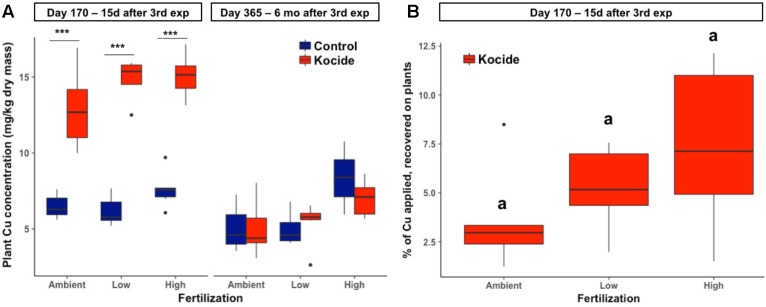
Effects of Kocide exposures and fertilizer treatments on plant biomass Cu concentrations on the two final harvest days (170 and 365): **(A)** Copper concentration associated to the aboveground plant biomass after the third Kocide exposure (170 days) and at the end of the experiment (365 days). **(B)** Percentage of the Cu applied as Kocide recovered associated to the aboveground plant biomass on day 170 (the Cu background measured in control plants was subtracted). In the **(A)**, significant effects of the Kocide treatment compared to the controls are indicated by asterisks: ^∗∗∗^*p* < 0.001 and the lack of significant differences between fertilization treatments in **(B)** are indicated by similar letters (a). The data are presented as box plots where the black horizontal line in the middle of the box represents the median and the two ends of the vertical line indicates the minimum and maximum values. The black dots represent the outliers.

Two weeks after the third and final Kocide exposure (day 170), plant biomass Cu concentrations increased twofold among the three fertilization treatments (*p* < 0.001, **Figure 6A**). Higher Cu concentration was found in the plant biomass of the High fertilization treatment compared to the Ambient fertilization in the mesocosms exposed to the Kocide (+17%, *p* = 0.03). Overall, plant biomass Cu concentrations on day 170 averaged at 14.3 ± 0.3 mg/kg in the Kocide-treated mesocosms, while the concentration was 6.75 ± 0.18 mg/kg in the Control mesocosms. Based on the known concentration of Cu applied as Kocide per mesocosm, we calculated the percentage of Cu recovered on the plants on day 170, and found that only a small fraction of the Cu applied to the mesocosms (3.7–7.4%) was associated to the plants 15 days after the last Kocide application (**Figure 6B**).

**FIGURE 7:**
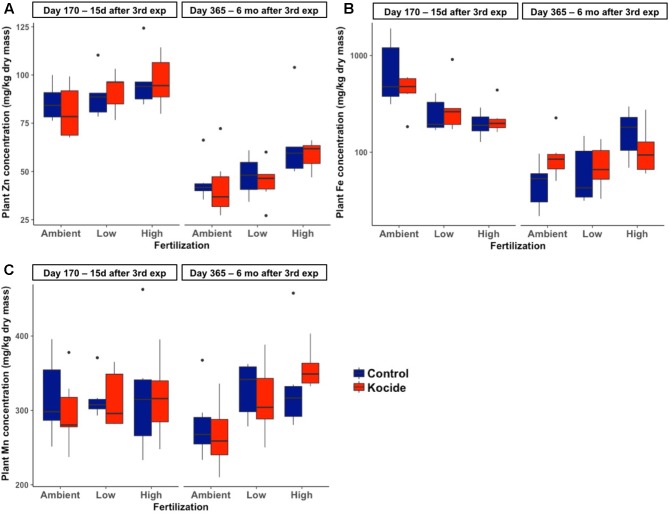
Effect of Kocide exposures in the different fertilization treatments on the mineral nutrient content of the aboveground biomass: **(A)** Zn, **(B)** Fe, and **(C)** Mn concentrations on days 170 and 365. Note that the *y*-axis of the **(B)** is on a log scale.

There was no effect of the fertilization treatment on the amount of Cu associated to aboveground plant biomass (fertilization effect; *p* = 0.16, **Figure 6B**) though it should be noted that the maximum Cu concentrations in biomass increased with increasing fertilization level. Six months after the last exposure, we found that the plant biomass Cu concentrations were no longer significantly different between control and Kocide-exposed mesocosms (day 365, **Figure 6A**). Moreover, on both final days (170 and 365), there were no significant changes in mineral nutrient content (incl. Zn, Mn, and Fe concentrations) of the plant biomass exposed to the Kocide exposures (**Figure 7**).

The soil Cu concentrations were not significantly different between the control and Kocide treatments, even immediately following the third Kocide exposure (day 170, *p* = 0.60). None of the soil Cu concentrations were significantly different from the high natural Cu background of this soil (90.5 ± 4.4 mg Cu/kg).

## Discussion

In our mesocosm experiment, repeated exposures to the nanopesticide Kocide 3000 had no negative effects on plant biomass and plant–microorganism associations but soil microbial enzyme activities were periodically inhibited or stimulated by this treatment. We observed interactive effects between the nanopesticide and the fertilization treatments leading to most of the detrimental effects found in the Ambient fertilization treatment.

### No Detrimental Effects on Plant and Plant–Microbe Associations

Other than a 14 and 27% increase on day 90 after the second exposure in the Low and High fertilization conditions, we found limited effects of the three Kocide applications on aboveground plant biomass. Previous work examining the effect of Kocide 3000 on plant biomass include reports of significant decreases in lettuce (Hong et al., 2015), elegant Clarkia (Conway et al., 2015), and maize (Zhao et al., 2017) shoot biomass (**Table 2**), as well as a report of lettuce leaf biomass stimulation (Zhao et al., 2016b). However, these studies were conducted in artificial growth media or potting soils with Kocide exposure in concentrations several orders of magnitude higher than the recommended doses (**Table 2**). By adhering to realistic Kocide concentrations and soil conditions, reflective of agricultural conditions, our results show that Kocide 3000 applications do not lead to any decrease in plant yields of a mixed forage plant community. In fact, we found that plant growth is stimulated when Kocide treatments are in tandem with fertilization. The fertilization may have alleviated stress such that any direct, negative impacts of Kocide may have been mediated. We hypothesize that the stimulations observed were probably related to direct effects of the nanopesticide on plant health or nutrition and not to an indirect positive effect driven by the microbial endpoints measured. When the plant biomass stimulations occurred (day 90), the six microbial enzyme activities tested were not significantly altered by the treatments, with the exception of a positive increase of the sulfatase activity in the High fertilization treatment.

**Table 2:** Comparison of the results of this study with previous published studies on the effects of the nanopesticide Kocide 3000 on crop biomass and microbial communities.

Reference	Concentration sprayed on plants or applied to soils	Medium	Crop	Duration	Effect on plant biomass	Cu concentration in plant biomass	Effect on microbial community
This study	Three applications of 6.68 mg/L at 2.5-month interval on plants	Sandy-clay-loam soil	Mixed forage	1 year	Increase of aboveground biomass in low (+14%) and high fertilization (+27%)	Aboveground biomass: 6–14 mg/kg	Inhibition or stimulation of microbial enzyme activities in the three fertilization treatments
Conway et al., 2015	1, 10, or 100 mg/L every week to soil	Potting soil	Herbaceous annual plant *Clarkia unguiculata*	8 weeks	Reduced growth rates, leaf production rates, and maximum number of leaves with increasing exposure concentrations in a low light – excess nutrient condition	Leaves: 80–800 mg/kg. Stems: 5–25 mg/kg	n.d
Hong et al., 2015	5, 10, or 20 mg/L in growth media	Hydro-ponics	Lettuce (*Lactuca sativa*) or alfalfa (*Medicago sativa*)	15 days	Reduced lettuce shoot length at 10 and 20 mg/L but no effect on alfalfa	Lettuce shoots: 20–52 mg/kg, Alfalfa shoots: 160–182 mg/kg	n.d
Zuverza-Mena et al., 2015	20 or 80 mg/kg in soil	Potting soil	Cilantro (*Coriandrum sativum*)	30 days	Increase of root biomass at the highest concentration and no effect of shoot biomass	Shoots: 10–15 mg/kg	n.d
Zhao et al., 2016a Nano Impact	1050 and 1555 mg/L two times per week on plants	Sandy-loam soil	Lettuce (*Lactuca sativa*)	30 days	n.d	Vascular tissues: 9.9, 823 and 1111 mg/kg, Photosynthetic tissues: 13.0, 1353 and 2008 mg/kg	n.d
Zhao et al., 2016b ES&T	1050, 1555, or 2100 mg/L two times per week on plants	Sandy-loam soil	Lettuce (*Lactuca sativa*)	30 days	Increase of leaf biomass at low and medium concentrations	Vascular tissues: 973–1344 mg/kg, Mesophyll tissues: 1695–2296 mg/kg	n.d
Zhao et al., 2017	100 or 1000 mg/L three times a day on plants	Artificial growth media	Maize (*Zea mays*)	7 days	The higher dose significantly decreased leaf biomass by 17-20%	Leaves: 12–1404 mg/kg	n.d


*n.d, not determined.*

Repeated Kocide applications did not alter mycorrhizal fungi colonization of plant roots, a finding consistent with reports demonstrating that non-nano copper-based fungicides, including copper oxychloride [Cu_2_(OH)_3_Cl, Sugavanam et al., 1994; Hernández-Dorrego and Mestre-Parés, 2010], Cu(OH)_2_ (Graham et al., 1986; Baijukya and Semu, 1998; Rutto et al., 2002), and copper sulfate (Nemec, 1980), do not impact mycorrhizal colonization. Overall, these results are consistent with the fact that mycorrhizal fungi have been found to be resistant to metal contamination and are involved in the alleviation of metal toxicity for plants, as evidenced by high mycorrhizal colonization rates in plants grown in metal-contaminated agricultural soils (Leyval et al., 1997; Jentschke and Godbold, 2000). Conversely, diazotrophs and especially the heterotrophic free-living bacteria involved in this process can exhibit a high sensitivity to metal pollution, including nanomaterial contamination (Giller et al., 1998; Judy et al., 2015; Chen et al., 2017). The lack of effect on N_2_ fixation rates in our experiment may be again related to the low nanopesticide concentrations applied to the system but also to the low abundance of legumes in the plant community and the resulting low N_2_ fixation rates measured, especially on day 365. Low N_2_ fixation rates may have also resulted from the lower soil moisture at this date than on day 170 (Supplementary Figure S2). Our experiment shows that realistic exposures to the Kocide nanopesticide under relevant agricultural practices, in outdoor conditions with a natural soil, do not lead to adverse effects on forage biomass and key plant–microorganism interactions.

### Interactive Effects Between Nanopesticide and Fertilization Treatments

The most sensitive endpoints to the Kocide exposures were the extracellular enzyme activities involved in OM degradation (C, N, P, S cycling) that are performed by a diverse group of soil microorganisms. Interestingly, the effects observed in the enzyme activities in the principal component analysis and when analyzing each enzyme separately were strongly influenced by the degree of fertilization and exposure duration. For instance, these significant interactions between the nanopesticide and fertilization treatments resulted in large decreases in all enzyme activities on the short-term after the first Kocide exposure in the Ambient fertilization, while three enzymes were decreased in the Low fertilization, and no effect was observed in the High fertilization condition. This result shows that—in a community of microorganisms not previously exposed to this nanopesticide—the resistance of microbial function to the nanopesticide increased with increasing soil nutrient availability. We hypothesize that the microbial communities involved in the enzyme synthesis in the Ambient or Low fertilization treatment were already stressed by nutrient limitation and thus had less energy to cope with the additional disturbance brought by the antimicrobial nanopesticide (e.g., less energy available for detoxification; Griffiths and Philippot, 2013).

However, this pattern associated with an increased resistance of microbial activity with increasing fertilization was clear only on the short-term (day 15). On subsequent sampling dates, the Kocide treatment generated no effects or stimulations of enzyme activities simultaneously in the Ambient and High fertilizations. Especially on the third exposure (day 155 and 170), the nanopesticide application was associated with significant increases in C, N, and S-degrading enzyme activities in both Ambient and High fertilization but not in the Low fertilization treatment. These treatment effects occurred at the end of the growing season in North Carolina (November). We hypothesize here that soil resources were more depleted than at previous dates (Supplementary Figure S2, soil NH_4_^+^ concentration) and that the Cu added along with other micronutrients included in the Kocide formulation may have stimulated some microbial activities. Supporting this hypothesis, we observed on day 170 and 365 that soil NH_4_^+^ concentration along with plant biomass were significantly correlated to the soil enzyme activity profiles on a principal component analysis. Additionally, the microbial community could have shifted to become more Cu tolerant after the two previous nanopesticide exposures through the exclusion of sensitive microbial taxa over time (Díaz-Raviña et al., 2007; Brandt et al., 2010).

Surprisingly, we observed large decreases of beta-glucosidase and phosphatase activities (-57 and -47%, respectively) on day 365, 6 months after the last Kocide application in Ambient fertilization conditions. These decreases occurred at the same period of the season that the initial enzyme activity inhibitions previously described on day 15 and may be related to microbial responses to Kocide driven by seasonal effects (e.g., lower water availability) or to legacy effects of the nanopesticide on the microbial community function. Our experiment was not able to confirm either of these hypotheses. Additional research on the long-term effects of chronic nanopesticide applications and the influence of season on plants and microorganisms’ responses to this agrochemical is needed.

Overall, the Kocide applications caused most of the inhibition on enzyme activities in the Ambient fertilization treatment and the majority of the positive effects on plant biomass and enzyme activities in the High fertilization treatment. These results suggest that when used in conventional farming with high fertilization rates, repeated Kocide 3000 application had limited negative consequences and induced positive effects on forage production and soil microbial processes over a growing season. However, in the context of lower-intensity fertilization where this nanopesticide is often used (e.g., organic farming), Kocide 3000 applications may have some unintended detrimental effects on microbially mediated soil processes involved in C and P cycling and on forage production.

### Limited Legacy Effects of Nanopesticide Applications After a Growing Season

Long-term contamination of agroecosystems with Cu fungicides used in both conventional and organic farming is of great environmental and toxicological concerns, as Cu has a low mobility in soil and can accumulate over time (Komárek et al., 2010; Navel and Martins, 2014). However, most previous studies examined Kocide exposure over very short temporal scales (up to 2 months), limiting realistic assessment of how Kocide may impact agroecosystems and non-target habitats in the long run. Additionally, these published experiments have been conducted in controlled laboratory and greenhouse conditions, where nutrients and water are not limiting, and in which the focus was plant-only, largely ignoring the potential impact on rhizospheric microbial communities (**Table 2**). Assessing the fate in soil and long-term non-target effects of the new generation of Cu nanopesticides under realistic conditions is necessary to determine if they should replace conventional pesticide formulations.

Following the last nanopesticide exposure performed in our experiment (day 170), we observed that the Cu concentration associated with aboveground plant biomass was double that of the control plants. This Cu accumulation was observed in all fertilization treatments and the Cu residue concentrations observed compared to the control plants were always lower than the recommended maximum residue levels authorized in the European Union market (10 mg Cu/kg residue, EU Pesticides Database). Copper concentrations in plant biomass observed in our study were 100 times lower than in previous publications simulating foliar applications of Kocide 3000 (**Table 2**) (Zhao et al., 2016a,b, 2017). In our study, less than 10% of the Cu applied during the Kocide application was recovered associated to the aboveground plant biomass after 15 days. This suggests that the majority of this nanopesticide ends up in the soils (93–97%), building up over time and that the amount of Cu exported during plant harvest is limited. In our experiment, we could not confirm that Cu accumulated on the soil surface because of the high natural Cu background concentration (90.5 ± 4.4 mg/kg) of our test soil compared to the low Cu amount applied to the mesocosms (total of 5.43 mg of Cu per mesocosm). Additionally, 6 months after stopping the nanopesticide exposures (day 365) we could not detect any differences in Cu associated with plants between control and Kocide-treated mesocosms. Taken together, these results show that three Kocide 3000 applications over a growing season led to no detectable legacy effects in terms of Cu accumulation in the plant and soil compartments 6 months later.

In terms of biological endpoints, the microbial enzyme activities were the only parameters showing significant declines 6 months after the last Kocide 3000 exposure. These legacy effects on beta-glucosidase and phosphatase activities are particularly interesting because these processes were not affected by Kocide additions shortly after the second and third exposures. This observation indicates that despite the high resilience of microbial processes on the short-term (day 75), longer term declines can occur and these phenomena are hard to predict based on our pre- and post-exposure monitoring during the growing season. These results call for long-term assessment of nanopesticide impact on soil fertility mediated by microbial processes to uncover the abiotic and microbial factors driving these declines. Additionally, in a parallel mesocosm experiment simulating a wetland ecosystem exposed chronically to Kocide 3000, we observed that this nanopesticide caused large ecosystem-scale impacts, including an intensification of eutrophication and major algal blooms (Simonin et al., 2018). Our findings demonstrate that this particular nanopesticide may have limited environmental consequences in the target terrestrial agroecosystems, but that non-target, downstream, aquatic ecosystems may be more vulnerable to impacts of nanopesticides in runoff.

## Author Contributions

MS, BC, SA, and EB designed the experiments. MS, SA, CB, and JR conducted the field work and the plant biomass and microbial enzyme analyses. WT and NC performed the N_2_ fixation assays. JJ performed the mycorrhizal colonization assays. JU performed the ICP-MS measurements. MS, SA, and EB wrote the paper and all the authors edited the manuscript.

## Conflict of Interest Statement

The authors declare that the research was conducted in the absence of any commercial or financial relationships that could be construed as a potential conflict of interest.

## Footnotes

1 https://content.ces.ncsu.edu/north-carolina-agricultural-chemicals-manual/fertilizer-use
