# Supplementary material for: Plant and Microbial Responses to Repeated Cu(OH)2 Nanopesticide Exposures Under Different Fertilization Levels in an Agro-Ecosystem
Source: Front Microbiol. 2018 Jul 31;9:1769. doi: 10.3389/fmicb.2018.01769 (PMC6079317; doi:10.3389/fmicb.2018.01769)
Supplement: Supplementary file 1 [file Table_1.DOCX]

**SUPPLEMENTARY INFORMATION**

Figure S1: Precipitation and average air temperature at the mesocosm site (WRCC-DRI Duke Forest Blackwood Division weather station, N 35° 58' 00", W 79° 05' 30") during the 365 days of the experiment.

Figure S2: Effect of Kocide exposures in the different fertilization treatments on A) soil moisture concentration and B) soil NH_4_^+^ concentration (no measurements on day 365). The points represent the means and the error bars are standard errors.
